# Coronary Artery Occlusion Detection Using 3-Lead ECG System Suitable for Credit Card-Size Personal Device Integration

**DOI:** 10.1016/j.jacadv.2023.100454

**Published:** 2023-07-27

**Authors:** Alexei Shvilkin, Dejan Vukajlović, Boško P. Bojović, Ljupčo R. Hadžievski, Branislav Vajdic, Vladimir A. Atanasoski, Marjan N. Miletić, Peter J. Zimetbaum, C. Michael Gibson, Vladan Vukčević

**Affiliations:** a Cardiovascular Division, Department of Medicine, Beth Israel Deaconess Medical Center, Boston, Massachusetts, USA; b Department of Medicine, Clinical Center of Serbia, Belgrade, Serbia; c Kliniken am Goldenen Steig, KH Grafenau, Kardiologie/Innere Medizin, Grafenau, Germany; d Vinča Institute of Nuclear Sciences, University of Belgrade, Belgrade, Serbia; e HeartBeam Inc, Santa Clara, California, USA

**Keywords:** acute coronary syndrome, electrocardiography, myocardial ischemia, ST-segment elevation myocardial infarction, vectorcardiography

## Abstract

**Background:**

Early coronary occlusion detection by portable personal device with limited number of electrocardiographic (ECG) leads might shorten symptom-to-balloon time in acute coronary syndromes.

**Objectives:**

The purpose of this study was to compare the accuracy of coronary occlusion detection using vectorcardgiographic analysis of a near-orthogonal 3-lead ECG configuration suitable for credit card-size personal device integration with automated and human 12 lead ECG interpretation.

**Methods:**

The 12-lead ECGs with 3 additional leads (“abc”) using 2 arm and 2 left parasternal electrodes were recorded in 66 patients undergoing percutaneous coronary intervention prior to (“baseline”, n = 66), immediately before (“preinflation”, n = 66), and after 90-second balloon coronary occlusion (“inflation”, n = 120). Performance of computer-measured ST-segment shift on vectorcardgiographic loops constructed from “abc” and 12 leads, standard 12-lead ECG, and consensus human interpretation in coronary occlusion detection were compared in “comparative” and “spot” modes (with/without reference to “baseline”) using areas under ROC curves (AUC), reliability, and sensitivity/specificity analysis.

**Results:**

Comparative “abc”-derived ST-segment shift was similar to two 12-lead methods (vector/traditional) in detecting balloon coronary occlusion (AUC = 0.95, 0.96, and 0.97, respectively, *P* = NS). Spot “abc” and 12-lead measurements (AUC = 0.72, 0.77, 0.68, respectively, *P* = NS) demonstrated poorer performance (*P* < 0.01 vs comparative measurements). Reliability analysis demonstrated comparative automated measurements in “good” agreement with reference (preinflation/inflation), while comparative human interpretation was in “moderate” range. Spot automated and human reading showed “poor” agreement.

**Conclusions:**

Vectorcardiographic ST-segment analysis using baseline comparison of 3-lead ECG system suitable for credit card-size personal device integration is similar to established 12-lead ECG methods in detecting balloon coronary occlusion.

Timely presentation of patients with ST-segment elevation myocardial infarction (STEMI) provides the best chances to minimize mortality and improve clinical outcomes.^1^, ^2^, ^3^ Prehospital delays constitute the major contributor to “symptoms to balloon” time with median symptoms to call time over 2.5 hours, by far exceeding in-hospital delays.^4^ Remote transmission of a prehospital ECG allows early identification of STEMI patients requiring emergent revascularization, which significantly reduces symptoms-to-balloon time and mortality^5^ and is a Class I recommendation for STEMI management.^6^

Prehospital 12-lead ECG recording requires large, nonportable, specialized equipment and trained personnel and may not be sufficiently efficient to minimize delays in the delivery of reperfusion. A single ECG tracing at one moment in time has limited accuracy in ischemia detection, while comparison of an ECG to a prior baseline ECG (comparative ECG analysis) significantly improves its accuracy.^7^

Attempts have been made to diagnose acute myocardial ischemia by using personal devices that do not require professional assistance with the goal to shorten prehospital delays in patients with acute coronary syndromes. A “multilead ECG equivalents” using sequential single lead recordings by portable devices (AliveCor and Apple Watch) were capable of myocardial infarction detection in emergency room (ER) patients.^8^^,^^9^ An implantable intracardiac ST-segment monitoring device with patient alert system decreased time to presentation in patients with acute coronary syndromes without increasing the number of unnecessary ER visits.^10^

Ischemia detection based upon a limited number of simultaneously recorded leads using vectorcardgiographic (VCG) analysis^11^^,^^12^ or a 12-lead reconstruction^13^ has been demonstrated to be feasible. A portable device with 3 wired electrodes suitable for self-application was superior to the standard 12-lead ECG criteria to detect myocardial ischemia using a set of asymptomatic recordings as baseline^14^ The presence of wires with disposable electrodes limits the utility of such device. ECG leads can be integrated into a portable device without the need for external electrodes/wires^13^ which would improve the ease of application and compliance.

## Objective of the study

To validate the accuracy of automated coronary occlusion detection during balloon coronary angioplasty using vectorcardiographic ST-segment analysis of a 3-lead near-orthogonal ECG system (“abc”) and compare it to the accuracy of the standard 12-lead ECG interpreted by automated computer analysis and expert human readers.

## Methods

The study was conducted at the Clinical Center of Serbia, Belgrade, Serbia, and was approved by the institutional Clinical Ethics Committee. All patients provided written consent.

### Patients

Consecutive patients (n = 66, men 79%, age 55 ± 9 years) with angiographically proven coronary artery disease (>70% diameter coronary artery stenosis) suitable for percutaneous coronary intervention (PCI) were enrolled. Patients were excluded if they had a pacemaker or implantable cardioverter-defibrillator, a STEMI within the past 7 days, left bundle branch block, allergic reaction to skin electrodes, or other conditions precluding completion of the study protocol.

#### PCI procedure description

Planned PCI was performed according to the standard Clinical Center of Serbia protocol. Transient coronary artery occlusion with the balloon catheter as part of the PCI procedure for a target duration of 90 seconds was used as a model of myocardial ischemia. Occlusion duration was shortened to the minimum of 60 seconds if patients developed severe chest pain, hemodynamic instability, or ventricular arrhythmias. When inflations were performed in more than one location, sufficient time was allowed between inflations to allow the ECG to return to baseline based on visual analysis. All patients were hemodynamically stable and chest pain-free prior to the catheterization.

### Electrocardiographic recordings

**Figure 1:**
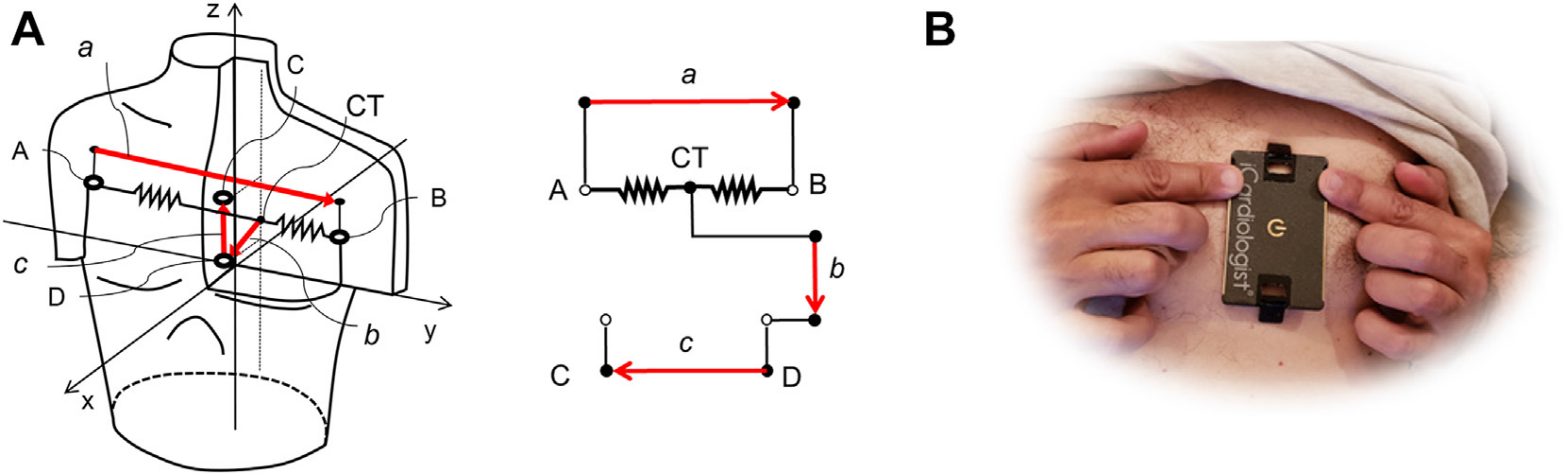
The “abc” Electrode System Electrode Positions and Electrical Schematic **(A)** Measured voltages: $V_{BA},V_{CA},V_{DA}$. Near-orthogonal “abc” leads: ${a=V}_{BA},b=V_{DA}-V_{BA}/2,c=V_{DA}-V_{CA}\text{.}$ Lead “a” is equivalent to the standard lead I (right to left arm) and similar to the orthogonal lead “x”, lead “b” has sagittal direction (back to front, similar to lead V_2_ or orthogonal “z”), lead c - caudal direction (head to toes, similar to lead aVF or orthogonal “y”). CT–central terminal. **(B)** Prototype HeartBeam credit card-size device with integrated leads “a”, “b”, and “c”. Chest electrodes unfold by a spring-loaded mechanism extending the intraelectrode distance

Standard 12-lead ECG with additional 4 electrodes in positions “A”, “B”, “C”, and “D” (Figure 1A) were digitally recorded continuously throughout the procedure using self-adhesive radiolucent wet electrodes (Quinton) and a 15-lead ECG recorder with standard specifications (500 Hz sampling rate, 0.05/150 Hz bandpass, and 50 Hz notch filters).

Electrodes A, B, C, D were designed to produce near-orthogonal leads “a”, “b”, “c” corresponding to the lead configuration that will be implemented in a portable device with integrated electrodes where right and left hand fingers produce lead “a” resembling the standard lead I similar to the commercially available ECG rhythm recorders, and together with the chest leads contribute to the vertical (“b”) and sagittal (“c”) leads (Figure 1B).

Because of the positional constraints during PCI procedure, fingertip electrodes to record lead “a” were substituted by electrodes A and B on the shoulders equivalent to standard lead I. Waveforms produced by this location of limb lead placement are equivalent to the classic wrist lead position.^15^

The following 10-second recording segments were used for analysis:

1. “Baseline” segment (n = 66): obtained in the cath lab holding area before the procedure;
2. “Preinflation” segment (n = 66): the patient positioned on the angiographic table prior to balloon inflation is considered negative reference (artery open);
3. “Inflation” segment (n = 120): ECG recording at the end of a 90-second balloon inflation, considered positive reference (artery occluded).

### ECG analysis

The initial portion of the ST-segment (starting at J point + 10 ms) was used to detect coronary artery occlusion (see Supplemental Methods for details). ST-segment analysis of the “abc” leads was performed using VCG approach in a manner similar to previously described in the literature^7^ treating leads “a”, “b”, and “c” as orthogonal (“a” = “x”, “b” = “z”, “c” = “y”) and compared to the standard 12-lead ECG analyzed in 4 different ways.

a) vectorcardiographic approach using leads “x”, “y”, “z” obtained by the Kors regression transformation of the 12-lead ECG^16^;
b) maximal single-lead ST-segment deviation measurement;
c) using the standard ischemia definition^17^;
d) consensus expert opinion.

All analyses except standard ischemia definition were performed in “spot” (single tracing) and “comparative” (comparison with “baseline” tracing) modes.

#### Vectorcardiographic measurement

Median QRST complex and QRST vector loops were constructed from 10-second signal segments using “abc” and 12-lead-derived “xyz” leads. Fiducial points were determined automatically by the custom-built software and confirmed by visual analysis.

The following measurements were constructed:

1. S_abc was calculated as the 3-dimensional abc-derived ST-segment vector loop segment deviation from isoelectric point averaged across the segment duration and measured in mV (“spot” measurement).
2. C_abc was calculated similarly by measuring the ST-segment vector loop segment difference between pairs of recordings: “baseline”-”preinflation” and “baseline”-”inflation” (“comparative” measurement).

Spot and comparative “xyz” measurements (S_xyz and C_xyz, respectively) were calculated in a similar manner using “xyz”-derived QRST vector loops.

The duration of the ST-segment portion used for all vector measurements was heart rate-corrected using linear dependence on the RR interval to minimize potential confounding effects of heart rate differences^18^; see Supplemental Methods for details.

**Figure 2:**
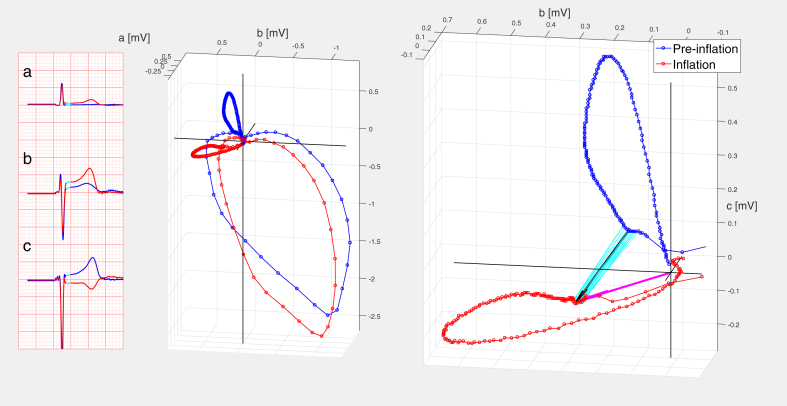
Calculation of “abc” Measurements C_abc and S_abc in a Patient With Proximal LAD Balloon Occlusion **(Left)** Median beat leads “a”, “b”, “c”. Cyan-colored portion of ST-segment used for calculation of diagnostic measurements. Middle **(Middle)** Modified left sagittal projection of the “abc”-based median QRST loops. **(Right)** Magnified “abc” T vector loops in the same projection. Cyan lines delineate analyzed ST-segment. **Black arrow length** represents the value of C_abc (=0.29 mV); **magenta arrow length** represents S_abc (=0.31 mV). Vector inscription velocity indicated by 2 ms inter-dot timing. **Blue** = preinflation; **red** = inflation. LAD = left anterior descending coronary artery.

Figure 2 illustrates S_abc and C_abc calculation in a patient with left anterior descending coronary artery (LAD) occlusion (corresponding 12 leads of “inflation” and “baseline” ECGs presented in Supplemental Figure 1).

#### 12-lead ECG measurements

Spot and comparative 12-lead-derived measurements S_12L and C_12L were calculated as the average ST-segment shift with segment duration and heart rate correction as above on the ECG lead with maximal ST-segment deviation with and without baseline subtraction, respectively.

Standard ischemia (ST-segment elevation and ST-segment depression) and STEMI (ST-segment elevation only) criteria were calculated based on the Fourth Universal Definition of Myocardial Infarction definition.^17^

### Human reading of the 12-lead ECG

Human 12-lead ECG interpretation was performed by 3 board-certified cardiologists (1 interventionalist, 2 electrophysiologists), designated as R1 to R3. The readers were not required to measure whether ST-segment shift conforms to the standard criteria for acute ischemia but rather deliver their expert opinion on whether the acute ischemia is present (Yes/No). Isolated T-wave inversions without concomitant ST-segment shift were not considered signs of ischemia.

Three separate readings were performed by each of the 3 readers R1 to R3.

1 “Human spot 1 (HS1)”: Blinded interpretation of single “preinflation” and “inflation” recordings.
2 “Human spot 2 (HS2)”: Repeat interpretation in the same fashion performed 2 to 3 months later to assess for intraobserver variability.
3 “Human comparative (HC)”: Interpretation of ECG pairs “baseline”-”preinflation” and “baseline”-”inflation” with “baseline” tracing explicitly marked.

All 12-lead ECG sets were presented to the readers in random order different for each reading.

For comparison with the automated methods of coronary occlusion detection, a consensus (“C”) human decision (agreed on by at least 2 readers) for each set of the above readings was used (HSC1, HSC2, HCC).

Separately, all “baseline” tracings were adjudicated visually for the presence of abnormalities such as pathologic Q waves, conduction defects, ST-segment shifts, and T-wave inversions >0.5 mm, confirmed by electronic waveform measurements.

Study procedures, signal recording, and interpretation sequence are presented in Supplemental Figure 2.

### Statistical analysis

Point and interval estimates for ST-segment shifts were calculated using general linear model analysis with fixed factors including coronary artery involved (LAD, left circumflex coronary artery [LCX], right coronary artery [RCA]), location of the inflated balloon within the artery (proximal, mid-, and distal), presence of baseline ECG abnormalities, and patients as random factor using Bonferroni correction for multiple comparisons. Correlation between measurement values was performed using Pearson’s correlation coefficient.

ROC curves were used to evaluate the performance of the automated measurements. Areas under the ROC curve (AUC) were compared using the DeLong method^19^ and 95% point confidence limits determined by bootstrapping. Optimal thresholds were determined from the ROC curves using Youden function and served to calculate the number of true positive/false positive/true negative/false negative recordings (TP, FP, TN, FN). Fisher exact test was used to compare sensitivity/specificity of the automated measurements and human binary decisions using 2 × 2 tables (TP/FP, TN/FN).

The validity (agreement with the reference) and reliability (inter- and intra-observer variability) of binary decisions was performed using interclass correlation coefficient (mixed model with absolute agreement), Spearman’s rho, and Krippendorf’s alpha (Kalpha) statistics.^20^, ^21^, ^22^ Two-sided *P* value of <0.05 was considered significant. All computations were performed using SPSS ver.20, IBM and MatLab ver. 2016b, MathWork).

## Results

### Baseline characteristics

A total of 66 patients were enrolled in the study. Patients’ clinical characteristics are presented in Table 1. The majority of patients (70%) were smokers, and 16 (24%) had prior myocardial infarctions. Only 15 out of 66 patients (23%) had “baseline” ECG interpreted as “normal”; 28 (42%) had ST-segment shift >0.5 mm in at least one of the leads.

**Table 1:** Clinical Characteristics of Patients

Sex M/F	52/14 (79/21)
Age, y	55.6 ± 9.4
Prior infarction	16 (24)
Diabetes	17 (24)
Hypertension	47 (71)
Hyperlipidemia	44 (67)
Smoking	46 (70)
Chronic kidney disease	24 (36)
BMI, kg/m^2^	26.9 ± 5.6
Prior PCI	3 (4.5)
Prior CABG	2 (3)
Diagnosis	
NSTEMI	3 (4.5)
Unstable angina	37 (56)
Sable angina	26 (39)
“Baseline” ECG	
Normal	15 (23)
Pathologic Q waves	26 (39)
Any lead S shift >0.5 mm	28 (42)
Any lead TWI >0.5 mm	27 (41)
Any lead S shift + TWI >0.5 mm	14 (21)

Values are n/n (%), mean ± SD, or n (%).

BMI = body mass index; CABG = coronary artery bypass grafting; ECG = electrocardiogram; NSTEMI = non-ST-segment elevation myocardial infarction; PCI = percutaneous coronary intervention; TWI = T wave inversion.

### Balloon occlusions

Balloon occlusion distribution and duration are presented in Table 2. There were 120 balloon occlusions performed. A total of 33 (50%) patients had angioplasty of more than 1 artery (range 1-6, median 2 per patient) (Supplemental Table 1). There was no difference in occlusion duration across the coronary arteries.

**Table 2:** Distribution of Balloon Inflations Per Artery

	LAD	LCX	RCA
Total	48 (40)	31 (26)	41 (34)
Proximal	19 (16)	10 (8)	19 (16)
Mid	22 (18)	14 (12)	15 (13)
Distal	7 (6)	7 (6)	7 (6)
Occlusion duration, s Mean (95% CI)	95 (91-98)	94 (90-99)	91 (87-95)

Values are n (%) unless otherwise indicated.

LAD = left anterior descending coronary artery; LCX = left circumflex coronary artery; RCA = right coronary artery.

### ECG analysis

There were 252 pairs (12-lead ECG + 3 “abc” leads) of signal recordings: 66 “baseline”, 66 “preinflation” and 120 “inflation” recordings corresponding to 120 balloon occlusions. Average heart rate remained unchanged between “baseline” and “preinflation” recordings. Heart rate response during coronary occlusion varied minimally by the artery with slight but significant difference between RCA and LAD occlusions (Supplemental Table 2).

Only 50 occlusions (42%) produced ST-segment changes reaching the standard ischemia criteria; among those, 39 occlusions (33%) produced ST-segment elevation matching the standard STEMI definition.

Balloon inflations resulted in significant ST-segment shift calculated by all measurements (Table 3). LAD inflations caused larger ST-segment changes compared to the LCX and RCA inflations. There were no significant differences in ST-segment shift observed between inflations in the proximal, mid-, and distal segments of any of the arteries by all methods of measurement.

**Table 3:** ST-Segment Measurements in “Preinflation” and “Inflation” Tracings by the Coronary Artery

Measurement	Optimal Threshold	“Preinflation”	“Inflation”^a^		
			LAD^b^	LCX	RCA
Comparative					
measurements					
C_abc	0.054	0.03 ± 0.01	0.19 ± 0.01	0.11 ± 0.01	0.13 ± 0.01
C_xyz	0.024	0.02 ± 0.01	0.12 ± 0.01	0.08 ± 0.01	0.10 ± 0.01
C_12L	0.047	0.03 ± 0.01	0.25 ± 0.01	0.12 ± 0.01	0.14 ± 0.01
Spot measurements					
S_abc	0.046	0.09 ± 0.01	0.24 ± 0.01	0.12 ± 0.02	0.13 ± 0.01
S_xyz	0.078	0.07 ± 0.01	0.17 ± 0.01	0.10 ± 0.01	0.12 ± 0.01
S_12L	0.064	0.13 ± 0.01	0.32 ± 0.01	0.15 ± 0.02	0.16 ± 0.01

Values are mean ± SEM.

LAD = left anterior descending coronary artery; LCX = left circumflex coronary artery; RCA = right coronary artery; SEM = standard error of the mean.

a *P* < 0.05 “Preinflation” vs “Inflation”, all measurements.

b *P* < 0.050 LAD vs LCX + RCA, all measurements.

### Occlusion detection performance of the automated measurements

**Figure 3:**
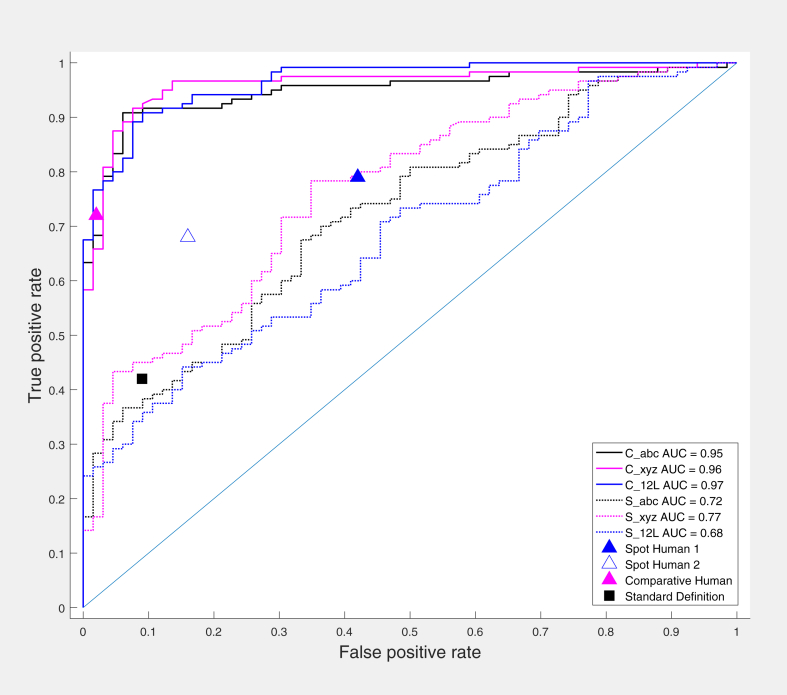
Automated Computer and Human Reader Performance ROC Curves AUC between comparative measurements were not different (*P* = NS) while significantly higher than AUC of spot measurements (*P* < 0.05). **Triangles** represent sensitivity/specificity data for consensus human readings (HCS1, HCS2, HCC); **black square** represents standard ischemia definition; **solid lines** represent comparative measurements; and **dashed/dotted lines** represent spot measurements. AUC = areas under the ROC curves

The performance of the automated measurements was evaluated using ROC curves. Areas under the ROC curves were compared, and optimal threshold points were determined (Table 3, Figure 3). Comparative measurements - C_abc, C_xyz, and C_12L (AUC = 0.948; 0.960; 0.965, respectively, *P* = NS) demonstrated near-uniform performance regardless of the artery, within-artery location, or presence of baseline ECG abnormalities with no significant differences between the measurements in any subset of tracings (Supplemental Table 3).

Spot measurements demonstrated much poorer performance (*P* < 0.05 compared to comparative ones) with no statistical differences between the individual measurements, S_abc, S_xyz, and S_12L (AUC 0.722; 0.766; 0.683, respectively, *P* = NS). The LAD distribution conferred numerically better, while LCX and the presence of the baseline ST-segment shift - numerically worse measurement performance, but the AUC differences did not reach statistical significance (Supplement Table 4).

### Correlation of comparative “abc” and 12-lead measurements

**Figure 4:**
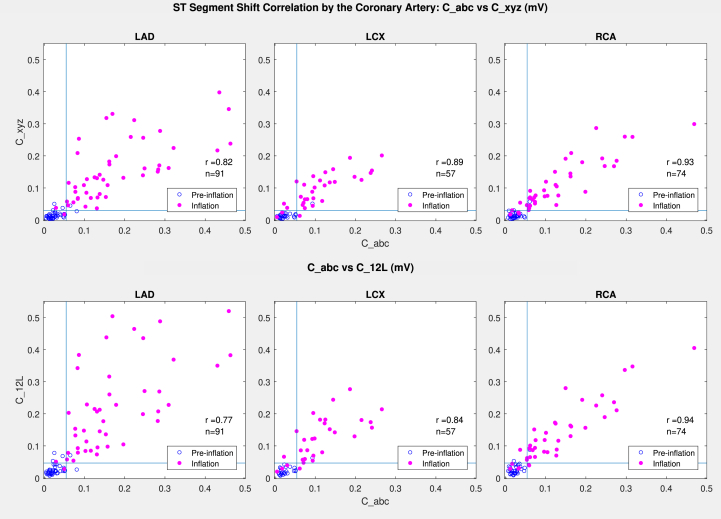
Correlation Between Comparative C_abc and 12 Lead-Derived (C_xyz and C_12L) ST-Segment Shift Measurements **Blue open circles** represent preinflation; **solid pink circles** represent inflation; and **blue lines** represent cutoff threshold values for respective measurements.

Numeric values of comparative “abc” lead-derived measurement C_abc demonstrated strong correlation with the 12-lead-derived comparative measurements (C_xyz, C_12L) in all arteries (r = 0.77-0.94) with 95% of readings being concordant (True/True, False/False), located in left lower/right upper quadrant marked by the cutoff threshold lines in Figure 4. Discordant readings were located close to the cutoff thresholds. As expected, the 12-lead-derived measurements C_xyz and C_12L demonstrated even stronger correlations (r = 0.96-0.97) between themselves (Supplemental Figure 3).

All automated “abc” and 12-lead measurements (both comparative and spot) correctly classified all tracings meeting standard STEMI and acute ischemia criteria (Supplemental Table 5).

To summarize, the 3-lead “abc” derived measurements performed similarly to the 2 12-lead measurements, “xyz” and “12L” (constructed with and without vectorcardiographic approach, respectively) in detecting coronary artery occlusion. All spot and comparative measurements correctly detected all STEMI tracings. Comparative measurements performed significantly better than spot ones.

### Human ECG interpretation

Human spot consensus interpretation (HCS1) in the whole group had sensitivity of 79% and specificity of 58%; comparative consensus interpretation (HCC) had sensitivity of 72% and specificity of 98%. The repeat human spot consensus interpretation (HCS2) used for reliability analysis had sensitivity of 68% and specificity of 83%, quite different from HSC1 with an increase in specificity and drop in sensitivity indicating high intraobserver variability (Figure 3). Human interpretation accuracy (both sensitivity and specificity) was the highest in the LAD territory, decreasing in RCA, and especially in LCX territory, where it was the worst (see Supplemental Table 6 for the detailed description of human and automated performance).

### Comparison of “abc” lead-derived measurements with human ECG interpretation

**Figure 5:**
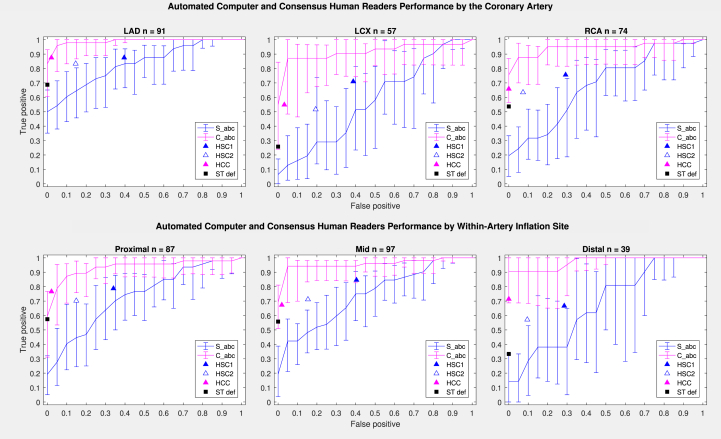
**“abc” Measurements and Human Reader Performance by the Occlusion Location ROC Curves** Error bars represent 95%-point confidence intervals. **Triangles** represent sensitivity/specificity data for consensus human readings (HSC1, HSC2, HCC); **blacksquare** represents standard ischemia definition; **magenta lines** represent comparative measurements; and **blue lines** represent spot measurements.

The comparative performance of automated measurements and human interpretation can be visualized by displaying the sensitivity/specificity values of the human readings along with the automated measurement ROC curves for the whole group (Figure 3), individual arterial distributions (Figure 5), and the presence of baseline ECG abnormalities (Supplemental Figure 4). The values for the consensus spot human interpretation (HSC1) in most artery locations projected below the ROC curves for the comparative measurement, C_abc, but above the ROC curves for the spot measurement, S_abc. Inclusion of baseline comparison in human comparative consensus interpretation (HCC) resulted in large improvement in specificity but slight drop in sensitivity, projecting on the steep ascending limb of the comparative automated measurements ROC curve far from optimal thresholds (Figures 3 and 5, Supplemental Figure 4).

### The accuracy of binary decisions comparison of automated measurements and human interpretations

The validity analysis (agreement with the reference—absence or presence of the balloon occlusion) of automated measurements and human readings using different criteria is presented in Table 4. Comparative automated measurements consistently demonstrated “good” agreement with reference (values >0.75), while comparative human reading (CHC) was in the “moderate” range (0.60-0.74). Spot automated measurements as well as spot consensus human readings (HSC1, HSC2) were in the “poor” range (values <0.50).^22^^,^^23^

**Table 4:** Validity Analysis: Agreement With Reference (Preocclusion/Occlusion)

Measurement	ICC	Rho	Kalpha
Comparative measurements			
C_abc	0.83 (0.78-0.93)	0.83 (0.74-0.91)	0.83 (0.70-0.94)
C_xyz	0.86 (0.82-0.89)	0.86 (0.77-0.93)	0.86 (0.74-0.96)
C_12L	0.81 (0.76-0.92)	0.81 (0.71-0.89)	0.80 (0.68-0.91)
Spot measurements^a^			
S_abc	0.33 (0.32-0.62)	0.33 (0.18-0.47)	0.32 (0.13-0.53)
S_xyz	0.25 (0.11-0.38)	0.29 (0.13-0.43)	0.22 (−0.16 to 0.45)
S_12L	0.23 (0.13-0.55)	0.31 (0.16-0.44)	0.16 (−0.10 to 0.41)
Human consensus reading			
HSC1	0.37 (0.24-0.49)	0.37 (0.23-0.50)	0.37 (0.19-0.56)
HSC2	0.47 (0.35-0.58)	0.49 (0.38-0.60)	0.46 (0.29-0.63)
CHC	0.63 (0.47-0.74)^b^	0.67 (0.60-0.76)	0.61 (0.45-0.77)

Values are mean (95% CI).

CHC = comparative human consensus reading; HSC = human spot consensus reading; ICC = interclass correlation coefficient.

a *P* < 0.05 spot vs comparative measurements.

b *P* < 0.05 vs C_abc.

### Reliability analysis of human interpretation

The estimates of human inter- and intra-observer variability are presented in Table 5. Overall interobserver reliability of human interpretation was only fair in spot readings HS1, HS2 (Kalpha = 0.52-0.60) and numerically improved by the presence of baseline comparison in the comparative reading, HC (Kalpha = 0.74), although not statistically significant. Only slightly more than half of the tracings (54% to 55%) were correctly classified by all readers R1 to R3 in HSC1 and HSC2, which increased to 68% when using baseline comparison (HCC). More than 10% of the tracings were misclassified by all three readers, even despite the presence of baseline for comparison.

**Table 5:** Inter observer and Intra observer Reliability of Human Readings

Interobserver	Kalpha, Mean (95% CI)	Number of Correctly Interpreted Tracings			
		By the Number of Readers (0-3)			
		3	2	1	0
HS1 (R1-R3)	0.52 (0.34-0.63)	101 (54)	34 (18)	30 (16)	21 (11)
HS2 (R1-R3)	0.60 (0.49-0.71)	103 (55)	34 (18)	21 (11)	28 (15)
HC (R1-R3)	0.74 (0.60-0.86)	127 (68)	24 (13)	12 (6)	23 (12)
		Number of tracings			
Intraobserver		Correctly Interpreted Number of Times (0-2) by Each Reader			
R1 (HS1 - HS2)	0.49 (0.40-0.58)		106 (57)	56 (30)	24 (13)
R2 (HS1 - HS2)	0.66 (0.53-0.77)		116 (62)	31 (17)	39 (21)
R3 (HS1 - HS2)	0.65 (0.53-0.76)		118 (63)	32 (17)	36 (19)

Values are n (%) unless otherwise indicated.

HC = human comparative; HS = human spot.

Intraobserver reliability was in the fair-to-good range with just above half of the tracings (57% to 63%) classified consistently correctly by the same reader. The number of tracings consistently misclassified in the spot mode by the same reader was between 13% and 21%.

## Discussion

The results of our study demonstrate that ECG recordings made with three “abc” leads can detect coronary occlusion comparable to the performance of a 12-lead ECG (Central Illustration). The 3-lead configuration in a mode looking for changes from baseline (comparative mode, C_abc) achieved over 90% sensitivity/specificity/accuracy in determining the state of the artery (open/closed), which is similar to other portable ECG recording technologies.^8^^,^^14^

**Central Illustration:**
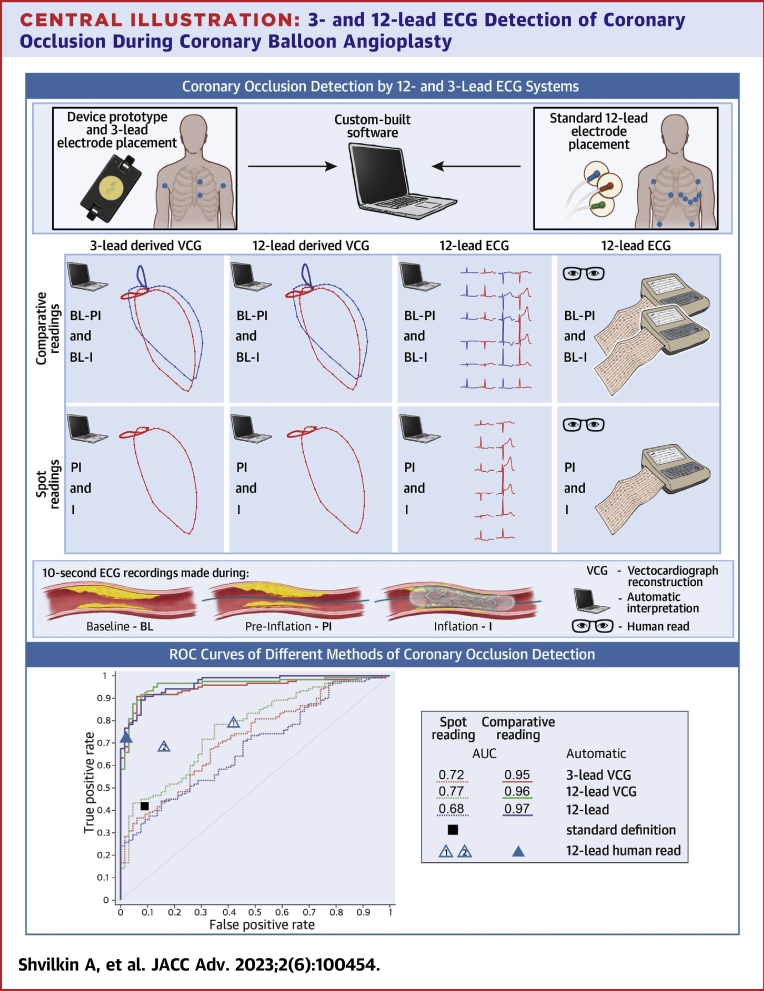
**3- and 12-lead ECG Detection of Coronary Occlusion During Coronary Balloon Angioplasty** ST-segment shift computer analysis for ischemia detection (inflation) with and without using vectorcardiographic reconstruction (VCG) and blind consensus human reading performed in “comparative” (baseline/inflation, baseline/preinflation comparison) and “spot” (preinflation, inflation without baseline information) modes. Areas under the ROC curves (AUC) along with points representing “STEMI standard definition” **(black square)** and human consensus readings (2 independent “spot”, **open triangles** 1 and 2, and 1 “comparative, **solid triangle**) are presented. All ECG analyses in “comparative” mode were superior to “spot” mode (*P* < 0.05 for AUC differences) without significant difference between “abc” and 12 leads. Human “spot” readings 1 and 2 highlight low reproducibility of human reading. ECG = electrocardiogram; STEMI = ST-segment elevation myocardial infarction

The advantage of the “abc” electrode configuration is that it can be incorporated into a credit card-size device without separate leads/wires with the limb lead “a” provided by direct finger to device contact improving its ease of use.

The information content of the 3 “abc” leads was comparable to that of the 12-lead ECG for the purpose of coronary occlusion detection based on the following findings: 1) there was no statistically significant difference between ROC curves of “abc” and 12-lead ECG-based comparative measurements; 2) coronary occlusion results in numerically similar changes in “abc”- and 12-lead-derived measurements that remain consistent across the occlusion locations and demonstrated close correlations; 3) binary decisions by “abc”- and 12-lead-derived comparative measurements demonstrate 95% concordance (eg, same true/false positive/negative cases) with no significant differences in validity analyses; 4) none of the measurements missed any tracings matching the standard ischemia definition.

The presence of a baseline nonischemic tracing gave a significant advantage to the comparative assessment over the assessment of ECG at a single point in time (spot assessment) in validity analyses. While comparative assessment resulted in “excellent/good” agreement, the spot assessment dropped to “poor-to-fair” agreement driven primarily by a decrease in specificity, which in turn was at least in part due to the high prevalence of baseline ECG abnormalities that are typical for patients presenting to the ER with chest pain.^24^ None of the spot assessment measurements missed any tracings meeting standard ischemia criteria, while the human readers missed between 1 and 3 such tracings even in comparative reading (Supplemental Table 5). Human ECG interpretation exhibited a significant amount of interobserver and intraobserver variability consistent with prior reports of wide variations in the accuracy of human ECG ischemia diagnosis.^25^^,^^26^ Only slightly over 50% of tracings were consistently interpreted correctly by the same reader during repeat reading, and between 11% and 15% of tracings were never interpreted correctly by any of the readers (Table 5).

A multicenter study of a portable 3-lead ECG device (with wired electrodes) employed a similar study design using 60-second coronary occlusions.^14^ The authors used a proprietary ST-segment analysis algorithm utilizing leads from the right shoulder to chest/right shoulder/left iliac crest and achieved overall sensitivity of 87%. In the present study, the methodology achieved a sensitivity of 92% using a more compact lead configuration suitable for integration into a credit card-size device. Another technology using a sequential smartphone-based single lead recordings to replicate a 12-lead ECG achieved sensitivity of 89% for STEMI and 84% for non-ST-segment elevation myocardial infarction among 204 ER chest pain patients as compared to the consensus human 12-lead ECG interpretation.^8^ These results indicate that the 3-lead “abc” ECG configuration performs similar to portable devices using wired leads.

The results of the present study highlight some issues pertinent to the development of portable ECG devices for myocardial ischemia detection.

1. The presence of a baseline recording improves diagnostic accuracy. Therefore, a portable ECG device will be more accurate when its use starts before the symptomatic event by establishing a personal baseline (preferably multiple recordings, for example up to 13 as advocated by Van Heuverswyn et al^14^), as opposed to using it as a portable ECG machine without the benefit of a baseline at a point of care location.
2. Isolated ST-segment-based ECG ischemia detection device will produce significant number of false positive results in low prevalence populations since ST-segment shift itself is not 100% specific for ischemia. As elegantly shown by Diamond and Forrester,^27^ the same degree of ST-segment depression of 0.5 to 1.0 mm during a stress test (similar to thresholds used in our study) portends the likelihood of coronary heart disease ranging from 0.3% in a 30-year-old asymptomatic woman to 94% in 60-year-old man with typical chest pain. Van Heuverswyn et al.^14^ observed a 4% false-positive rate among over 5000 asymptomatic outpatient recordings. Therefore, any ECG-based test for ischemia detection should include consideration of the pretest probability. Further studies will be needed to develop these methods.

### Study Limitations

In this proof of principle study, we used traditional ECG electrodes instead of integrated dry ones. Using wet electrodes might have decreased the level of noise and thus artificially improved results compared to the dry electrode design. All recordings were performed in close temporal proximity in supine patient position, minimizing the effects of temporal variations in heart rate, body position, and potential spontaneous ECG changes over time. Occlusion duration was limited by the short time of balloon inflation. Therefore, ischemic ECG changes evolving over longer time could not be captured. This could affect the “abc” lead performance in real world and could have artificially decreased the human readers’ performance. Balloon inflation was arbitrarily considered as a positive reference for ischemia. It is likely that some of the inflations did not produce ischemic ECG changes due to the presence of extensive collaterals, small area of ischemia, or pre-existing infarction in the artery distribution. All patients had known coronary heart disease, and ST-segment deviation was considered specific sign of ischemia. In unselected populations, there are multiple causes of nonischemic ST-segment changes that can result in false-positive ischemia detection especially in low prevalence groups.^24^^,^^27^ There were no patients with left bundle branch block in the study; the performance of “abc” lead measurements in this population is unknown. In this study, we did not include patients with ambulatory asymptomatic recordings to assess the rate of false-positive results in real life.

## Conclusions

Vectorcardiographic analysis of 3-lead “abc” ECG system detects coronary artery occlusion during balloon inflation, similar to the traditional 12-lead ECG. More research is needed to determine whether this method may be useful in the detection of myocardial ischemia in clinical settings.

PERSPECTIVES **COMPETENCY IN CLINICAL CARE AND PROCEDURAL SKILLS:** Coronary artery occlusion detection is possible using automated VCG analysis of a limited number of ECG leads suitable for implementation in a handheld portable device without wire electrodes. Further studies are needed to evaluate “abc” lead system performance in real-life clinical scenarios.

**TRANSLATIONAL OUTLOOK:** Clinical trials using portable device with VCG analysis of limited number of ECG leads will help establish its benefit on clinical patient outcomes.

## Funding support and author disclosures

Drs Shvilkin, Vukčević, Vukajlović, Bojović, Hadžievski, and Atanasoskiare are stockholders of HeartBeam, Inc. Drs Miletić, Zimetbaum, and Gibson have received consulting fees from HeartBeam, Inc. Dr Vajdic is CEO, HeartBeam, Inc.
