# Supplementary material for: Coronary Artery Occlusion Detection Using 3-Lead ECG System Suitable for Credit Card-Size Personal Device Integration
Source: JACC Adv. 2023 Jul 27;2(6):100454. doi: 10.1016/j.jacadv.2023.100454 (PMC11198085; doi:10.1016/j.jacadv.2023.100454)
Supplement: Supplement Figures 1-4 and Tables — 1-6 [file mmc1.docx]

**SUPPLEMENTAL APPENDIX**

**SupplementalMethods**

**Formulas used for vectorcardiography-based measurements:**

ST segment duration used for analysis determined as [J+10 to J+ dT] where

dT=[0.06s-(0.117/4)*(1-RR)]*1000, where RR interval is expressed in seconds and dT in milliseconds.

ST vector is defined as

$$\vec{S}_{\mathrm{xyz}}=X_{st}\vec{i}+Y_{st}\vec{j}+ Z_{st}\vec{k}$$

where

$X_{st}$,$Y_{st}$, $Z_{st}$ are average voltages of the signals X, Y, Z in the interval (J +10 ms) to (J+dT).

$S_{\mathrm{xyz}}$ is the magnitude of the vector $\vec{S}_{\mathrm{xyz}}$

$$S_{\mathrm{xyz}}=\sqrt{X_{st}^{2}+Y_{st}^{2} +Z_{st}^{2}}$$

$\vec{C}_{\mathrm{xyz}}$ is the vector difference between vector $\vec{S}_{\mathrm{xyz}}$ during the Inflation (${\vec{\mathrm{Si}}}_{\mathrm{xyz}}),$or Pre-Inflation (${\vec{\mathrm{Sp}}}_{\mathrm{xyz}}),$ and Baseline ${\vec{\mathrm{Sb}}}_{\mathrm{xyz}}$recordings

$$\vec{C}_{\mathrm{xyz}}={\vec{\mathrm{Si}}}_{\mathrm{xyz}}-{\vec{\mathrm{Sb}}}_{\mathrm{xyz}}=({Xi}_{st}-{Xb}_{st})\vec{i}+({Yi}_{st}-{Yb}_{st})\vec{j}+ ({Zi}_{st}-{Zb}_{st})\vec{k}$$

where

${Xi}_{st}$,${Yi}_{st}$, ${Zi}_{st}$ are average voltages of the signals X, Y, Z in the interval (J +10 ms) to (J+dT) of the recordings during the Inflation

${Xb}_{st}$,${Yb}_{st}$, ${Zb}_{st}$ are average voltages of the signals X, Y, Z in the interval (J +10 ms) to (J+dT) of the the baseline recordings

$C_{\mathrm{xyz}}$ is the magnitude of the vector $\vec{C}_{\mathrm{xyz}}$

$$C_{\mathrm{xyz}} =\sqrt{({Xi}_{st}-{Xb}_{st})^{2}+({Yi}_{st}-{Yb}_{st})^{2}+ ({Zi}_{st}-{Zb}_{st})^{2}}$$

**Supplemental Table 1.** Number of balloon inflations per patient

| Number of inflations per patient | Number of patients, n (%) |
| --- | --- |
| 1 | 33 (50) |
| 2 | 19 (29) |
| 3 | 10 (15) |
| 4 | 2 (3) |
| 5 | 1 (1.5) |
| 6 | 1 (1.5) |

**Supplemental Table 2**. Heart rate

|  | “Baseline” | “Pre-inflation” | “Inflation” | | |
| --- | --- | --- | --- | --- | --- |
|  |  |  | LAD | LCX | RCA |
| Heart Rate, bpm  Mean (95% CI) | 71 (69 – 74) | 70 (67 – 72) | 72 (68-76) | 70 (64-76) | 67 (63-70)* |
| * p <0.05 RCA vs LAD | | | | | |

**Supplemental Table 3.** Comparative measurements AUC by the artery, within-artery location, and presence of baseline ECG abnormalities (Means, 95% CI), p = ns.

|  | “Pre-inflation” n | “Inflation” n | C_abc | C_xyz | C_12L |
| --- | --- | --- | --- | --- | --- |
| All tracings | 66 | 120 | 0.948 0.906-0.972 | 0.960 0.921-0.982 | 0.965 0.935-0.982 |
| Coronary artery | | | | | |
| LAD | 43 | 48 | 0.987 0.946-0.998 | 0.990 0.959-0.998 | 0.989 0.963-0.998 |
| LCX | 26 | 31 | 0.912 0.785-0.971 | 0.955 0.857-0.991 | 0.959 0.823-0.990 |
| RCA | 33 | 41 | 0.945 0.857-0.983 | 0.980 0.928-0.996 | 0.957 0.907-0.985 |
| Within-artery location | | | | | |
| Proximal | 40 | 47 | 0.931 0.850-0.972 | 0.952 9.880-0.981 | 0.944 0.881-0.977 |
| Mid | 45 | 52 | 0.954 0.886-0.984 | 0.922 0.9700-0.998 | 0.990 0.963-0.998 |
| Distal | 18 | 21 | 0.971 0.863-1,000 | 0.989 0.934-1.000 | 0.968 0.858-0.997 |
| “Baseline” ECG | | | | | |
| Normal | 15 | 22 | 0.964 0.835-1.000 | 1.000 | 0.994 0.938-1.000 |
| ST shift > 0.05 mV | 28 | 50 | 0.937 0.905-0.989 | 0.984 0.942-0.996 | 0.975 0.929-0.995 |
| T wave inversion > 0.05 mV | 27 | 52 | 0.973 0.912-0.994 | 0.984 0.942-0.997 | 0.976 0.929-0.995 |
| ST shift + TWI | 14 | 21 | 0.971 0.870-1.000 | 1.000 | 0.989 0.931-1.000 |

**Supplemental Table 4.** Spot measurements AUC by the artery, within-artery location, and presence of baseline ECG abnormalities (Means, 95% CI), p = ns.

|  | “Baseline” n | “Ischemia” n | S_abc | S_xyz | S_12L |
| --- | --- | --- | --- | --- | --- |
| All patients | 66 | 120 | 0.722 0.637-0.787 | 0.766 0.681-0.826 | 0.683 0.602-0.755 |
| Coronary artery | | | | | |
| LAD | 43 | 48 | 0.832 0.736-0.904 | 0.857 0.761-0.926 | 0.838 0.726-0.905 |
| LCX | 26 | 31 | 0.568 0.417-0.712 | 0.467 0.304-0.642 | 0.473 0.326-0.633 |
| RCA | 33 | 41 | 0.693 0.551-0.801 | 0.687 0.550-0.0.804 | 0.657 0.518-0.766 |
| Within-artery location | | | | | |
| Proximal | 40 | 47 | 0.725 0.607-0.820 | 0.727 0.612-0.820 | 0.677 0.566-0.773 |
| Mid | 45 | 52 | 0.742 0.636-0.826 | 0.714 0.603-0.808 | 0.709 0.600-0.807 |
| Distal | 18 | 21 | 0.672 0.404-0.829 | 0.624 0.400-0.789 | 0.598 0.388-0.780 |
| Baseline ECG | | | | | |
| Normal | 15 | 22 | 0.724 0.534-0.876 | 0.749 0.599-0.907 | 0.712 0.505-0.846 |
| ST shift > 0.05 mV | 28 | 50 | 0.677 0.540-0.791 | 0.641 0.508-0.762 | 0.672 0.547-0.789 |
| TWI > 0.05 mV | 27 | 52 | 0.753 0.632-0.847 | 0.723 0.608-0.833 | 0.747 0.630-0.848 |
| ST shift + TWI | 14 | 21 | 0.650 0.450-0.806 | 0.622 0.440- 0.768 | 0.661 0.474-0.816 |

**Supplemental Table 5.** Binary decision performance in coronary occlusion detection by the Standard criteria (number of false negative cases)

|  | Standard Ischemia definition | |
| --- | --- | --- |
| Marker/reading | ST Depression (n = 11) | STEMI (n=39) |
| S_abc | 0 | 0 |
| C_abc | 0 | 0 |
| S_xyz | 0 | 0 |
| C_xyz | 0 | 0 |
| S_12L | 0 | 0 |
| C_12L | 0 | 0 |
| HSC1 | 0 | 1 |
| HSC2 | 1 | 2 |
| HCC | 1 | 2 |

**Supplemental Table 6.** True/False Positive/Negative and Hit/Miss counts; Sensitivity, Specificity, and Accuracy of binary decisions by “abc” measurements, Consensus Human Reading, and Standard Ischemia definition.

| Marker | “Inflation” tracings | | “Pre-inflation” tracings | | Sens  % | Spec  % | “Hit” (TP+TN) n | “Miss” (FP+FN) n | Accuracy % |
| --- | --- | --- | --- | --- | --- | --- | --- | --- | --- |
|  | TP | FN | TN | FP |  |  |  |  |  |
| **All records** | N = 120 | | N = 66 | | Total = 186 | | | | |
| C_abc | 109 | 11 | 62 | 4 | 91* | 94* | 171 | 15 | 92* |
| S_abc | 96 | 24 | 34 | 32 | 80§ | 52† | 130 | 56 | 70‡ |
| HSC1 | 95 | 25 | 38 | 28 | 79 | 58 | 133 | 53 | 72 |
| HSC2 | 82 | 38 | 55 | 11 | 68 | 84 | 137 | 49 | 74 |
| HCC | 86 | 34 | 65 | 1 | 72 | 98 | 151 | 35 | 81 |
| Standard | 50 | 70 | 62 | 4 | 42 | 91 | 112 | 74 | 60 |
| **LAD** | N = 48 | | N = 43 | | Total = 91 | | | | |
| C_abc | 46 | 2 | 41 | 2 | 96 § | 95 | 87 | 4 | 96^?^ |
| S_abc | 43 | 5 | 22 | 21 | 90§ | 51 | 64 | 27 | 70† |
| HSC1 | 42 | 6 | 24 | 19 | 88 | 56 | 66 | 25 | 73 |
| HSC2 | 40 | 8 | 36 | 7 | 83 | 84 | 76 | 15 | 84 |
| HCC | 42 | 6 | 42 | 1 | 88 | 98 | 84 | 7 | 92 |
| Standard | 30 | 18 | 41 | 2 | 63 | 95 | 71 | 20 | 78 |
| **LCX** | N = 31 | | n = 26 | | Total = 57 | | | | |
| C_abc | 27 | 4 | 25 | 1 | 87* | 96 | 52 | 5 | 91* |
| S_abc | 22 | 9 | 12 | 14 | 71§ | 46 | 34 | 23 | 60 |
| HSC1 | 22 | 9 | 14 | 12 | 71 | 54 | 36 | 21 | 63 |
| HSC2 | 15 | 16 | 20 | 6 | 48 | 77 | 36 | 21 | 63 |
| HCC | 17 | 14 | 25 | 1 | 55 | 96 | 42 | 15 | 74 |
| Standard | 6 | 25 | 24 | 1 | 19 | 92 | 30 | 26 | 53 |
| **RCA** | N = 41 | | n = 33 | | Total = 74 | | | | |
| C_abc | 36 | 5 | 32 | 1 | 88§ | 97 | 68 | 6 | 92* |
| S_abc | 32 | 9 | 18 | 15 | 78§ | 55 | 47 | 27 | 64‡ |
| HSC1 | 31 | 10 | 21 | 12 | 76 | 64 | 52 | 22 | 70 |
| HSC2 | 26 | 15 | 30 | 3 | 63 | 91 | 56 | 18 | 76 |
| HCC | 27 | 14 | 33 | 0 | 66 | 100 | 60 | 14 | 81 |
| Standard | 14 | 27 | 31 | 2 | 34 | 94 | 45 | 29 | 61 |
| p<0.05:  * higher than the all other; † lower than HSC2, HCC; ‡ - lower than HCC; § - higher than Standard; ? - higher than HSC1, HSC2, Standard | | | | | | | | | |

**Supplemental Figure 1.**A) 12-Lead ECG of a patient in Figure 2 during proximal LAD balloon occlusion (“Inflation”)B) “Baseline” ECG of the same patient

**Supplemental Figure 2**

Study procedures.

“Baseline” recordings (12L ECG and “abc” leads) obtained in the cath lab holding area. “Pre-inflation” recordings obtained in the cath lab immediately prior to the balloon positioning in the coronary artery. “Inflation” recordings obtained after 90 second balloon inflation. Spot computer ECG measurements and human reading performed on single tracings; Comparative computer measurements and human readings obtained by comparison with the “Baseline” tracing.


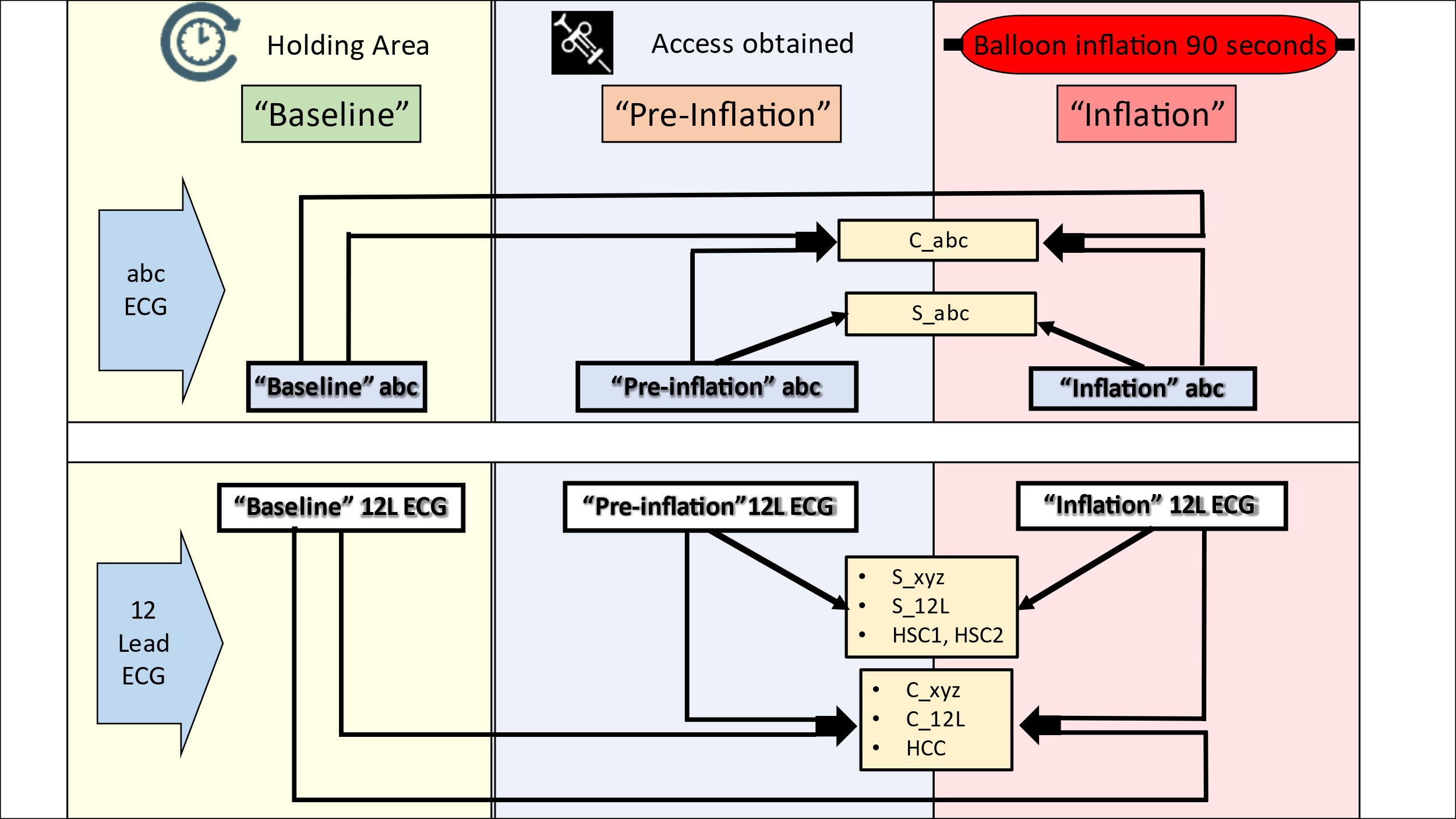


**Supplemental Figure 3**

Correlation between Comparative C_xyz and C_12L ECG measurements.

Top row: Breakdown by the coronary artery; Bottom row: Breakdown by location. Blue lines represent cutoff threshold values for respective measurements.

**Supplement Figure 4.**

“abc” measurements and human reader performance by the presence of baseline ECG abnormalities.

ROC curves: magenta lines represent Comparative and blue– Spot measurements. Error bars represent 95%-point confidence intervals. Triangles represent Sensitivity/Specificity data for Consensus human readings (HSC1, HSC2, HCC), black square – Standard ischemia definition.
